# Exploration of the Challenges of COVID-19 from the Perspective of Emergency Medicine Specialists

**DOI:** 10.1155/2024/5536103

**Published:** 2024-05-25

**Authors:** Tayebeh Rakhshani, Farzaneh Ghalehgolab, Mohammad Amin Bahrami, Shahnaz Karimi, Hadid Hamrah, Fatemeh Jafari, Ali Khani Jeihooni

**Affiliations:** ^1^Nutrition Research Center, Department of Public Health, School of Health, Shiraz University of Medical Sciences, Shiraz, Iran; ^2^Student Research Committee, Shiraz University of Medical Sciences, Shiraz, Iran; ^3^Department of Health Services Management, School of Management and Medical Informatics, Shiraz University of Medical Sciences, Shiraz, Iran; ^4^Department of Nursing, School of Nursing, Fasa University of Medical Sciences, Fasa, Iran; ^5^Department of Emergency Medicine, School of Medicine, Shiraz University of Medical Sciences, Shiraz, Iran; ^6^Department of Epidemiology, School of Health, Shiraz University of Medical Sciences, Shiraz, Iran; ^7^Nutrition Research Center, Department of Public Health, School of Health, Shiraz University of Medical Sciences, Shiraz, Iran

## Abstract

**Background:**

Emergency physicians are at the forefront of the medical system in the face of the COVID-19 crisis. Identifying the challenges, along with the strategies and effective measures implemented by them in the face of the COVID-19 crisis, can be a roadmap for future crisis management planning. This study aims to explain the challenges faced by emergency physicians regarding COVID-19.

**Methods:**

This study is a qualitative content analysis. Data were collected using individual and semistructured interviews. Twenty-seven emergency medical specialists in Fars University of Medical Sciences, Iran, participated in the study by purposive sampling method and were interviewed using semistructured interviews.

**Results:**

Participants' experience of COVID-19 led to the extraction of four main themes, including structural factors, threats to the health of the medical team, fluctuations of extremism and wastage in the face of COVID-19, and the country's policymaking hierarchy.

**Conclusions:**

Emergency physicians face challenges such as structural factors, health threats, extreme fluctuations, and national policymaking. To avoid surprises and threats, they must predict acute scenarios, provide necessary equipment, address skilled manpower shortages, and adopt appropriate management policies. This includes culture-building, cross-sector coordination, planning, and efficient management to prevent virus spread.

## 1. Introduction

The COVID-19 pandemic caused by severe acute coronavirus syndrome-2 (SARS-CoV-2) originated in Wuhan, China, in December 2019 and spread worldwide [1]. The World Health Organization declared the spread of COVID-19 in more than 100 countries (most prevalent in the United States, Italy, China, Spain, Germany, Iran, and France) on March 29, 2020, and it received international attention as a public health emergency [2, 3]. Countries with a shortage of health care equipment were at higher risk for the disease [4]. According to the World Health Organization, the total number of definite cases of COVID-19 in the world was 150989419 until May 2, 2021, and the number of deaths was 3173576 as well [5].

While being at risk of infection, medical staff face other problems, including burnout, difficult triage decisions, separation from family, stigma, and the pain of losing colleagues [6], along with increased workload, increased working hours, and frequent exposure to the suffering and death of patients [7]. Although the clinical manifestations and epidemiological characteristics of the virus have received considerable attention, there is still a notable gap in comprehensively addressing the unique challenges faced by frontline health care providers. Existing literature tends to focus on broader health care systems' responses, public health measures, and clinical management protocols, often overlooking the nuanced experiences and perspectives of those directly involved in patient care at the frontline. Consequently, there is a need for deep research to delve into the specific challenges, stressors, and coping mechanisms of emergency medicine specialists amidst the ongoing pandemic.

Considering the importance of COVID-19, the increasing prevalence of the disease, and the role of health workers, especially emergency medicine specialists, in dealing with coronavirus, and the lack of sufficient information about the impact of epidemics on emergency medical services (EMS) activity patterns [8, 9], this study was carried out to explain the challenges faced by emergency medicine specialists during COVID-19. We hope that this study will effectively bring the attention of health policymakers to the suffering and problems of health workers, enhance their quality of life during the pandemic, and enable them to provide better health services and manage the pandemic more efficiently.

## 2. Methods

This study is a qualitative content analysis. The research environment was Fars University Medical Sciences, Iran, and the samples included 27 emergency medicine specialists involved in the treatment of COVID-19 patients and the challenges of dealing with the spread of the disease. All of the emergency medicine specialists worked well and were equally exposed to the disease during the pandemic.

We collected the data through interviews conducted during the COVID-19 pandemic outbreak.

The inclusion criteria included willingness and interest in participating in research and sharing information and experiences. Furthermore, having at least 5 months of experience in treating COVID-19 patients and at least 5 years of experience as an emergency medicine specialist was the main condition for participation in the study.

We used the purposive sampling method in this study to select samples from emergency medicine specialists. Given that qualitative research prioritizes information from situations or events over sample size, we continued sampling through interviews until we reached data saturation. The interviews were semistructured, and since some aspects of exploring the challenges of facing COVID-19 were unknown, not all questions were prepared in advance but were designed during the study process.

The place of the interviews was the specialists' offices, and the time of the interviews was determined by agreement. In this study, the following ethical considerations were observed: obtaining the subjects' informed consent, explaining the research objectives before each interview, the reason for recording the interviews, confidentiality of the interviewees' identities, and the subjects' right to ask for the tape and the transcripts. The interviews (both in person and over the telephone) were recorded in full. They were then transcribed and typed.

Given that in qualitative research, interview questions play a very important role and semistructured interviews guide qualitative research, and considering that the questions in the present research were related to COVID-19 management challenges, they were designed, completed, and modified by the research team and qualitative research experts. We used the following set of questions to determine the challenges faced by emergency medicine specialists in dealing with COVID-19.

1. Describe one of your workdays dealing with COVID-19.
2. What challenges do you face in dealing with COVID-19 patients?
3. What factors contributed to the emergence of these challenges?
4. Who is involved in the challenges?
5. How did you or your patients feel when the challenges arose?
6. What are the outcomes of the challenges for you, the patients, and the health system?
7. What conditions or factors can help address the existing challenges?
8. What solutions do you suggest to address the existing challenges?
9. What are the strengths and weaknesses of the emergency medicine curriculum for emerging diseases?

Clearly, we asked more exploratory questions in response to the participants' answers.

The data were analyzed using qualitative inductive content analysis. It started right after the first interview, and the researcher started coding and classification after the first two ones. We used the MAXQDA 10 software to analyze the interviews. Classification and naming of the classes were done under the supervision of an observer experienced in qualitative data analysis, the main themes of the study were extracted, and the relationships between the classes were identified. We used peer review and member-checking methods to ensure that the data interpretation accurately reflected the phenomenon under study. To this end, the results of the data analysis and classification were provided to three university professors who were familiar with qualitative research and data analysis.

## 3. Results

We studied a total of 27 emergency medicine specialists, aged 32 to 60, with 11 (40.74%) females and 16 (59.26%) males. The participants were emergency medicine specialists with 5 to 20 years of clinical experience in the emergency department.

The participants' experiences with COVID-19 led to the extraction of four main themes, including structural factors, threats to the health of medical teams, fluctuations of extremism and wastage in the face of COVID-19, and policymaking at the national level. These classes consisted of 12 subclasses (Table 1), as described below.

### 3.1. Structural Factors

The first major class was structural factors, which consisted of three subclasses. At the early stages of dealing with patients with COVID-19 and at the peak of the disease, emergency physicians faced structural challenges such as a lack of medical equipment, a shortage of human forces, and a lack of physical space for patient hospitalization.

### 3.2. Deficit of Medical Equipment

The participants experienced problems such as old oxygen generators, a lack of perfect CT scanners to use all day, inadequate protective equipment, a lack of medication, and a lack of ventilators. The unavailability of required equipment wasted time, caused delays, and interrupted patient care. However, due to the advancement of medical science and equipment, the facilities and infrastructure of the emergency department should be updated so that patients have relative peace until the end of their treatment, and the hospital environment and lack of facilities should not add to their stress.

“The lack of necessary equipment and facilities causes anxiety and discomfort both for physicians and patients.”

“One of the challenges we faced early on was not having a ventilator, not having a monitoring bed, and not having proper devices (M5).”

#### 3.2.1. Insufficient Hospitalization Space

According to the participants' experiences, the physical space conditions in hospitals became more complicated during the peak of the disease and were not adequate for patient hospitalization. No unoccupied bed was available for patient admission, and both positive and suspicious cases of COVID-19 shared the same environment. There were no suitable conditions to separate COVID and non-COVID patients' bathrooms.

“The lack of enough space makes it impossible to distance admitted patients from each other.”

#### 3.2.2. Shortage of Human Forces

Based on the participants' experiences, there was no appropriate ratio between hospital beds and human forces in hospitals, and this significantly increased the workload of the physicians and nurses in COVID-19 wards. As the participants stated, although new personnel were used in COVID-19 wards to solve the problem of human force shortages, experienced personnel were needed in these wards.

“Newly graduated students are now employed in COVID wards. They aren't experienced enough to work in this clinical setting and don't act as fast as they ought to.”

“There was a shortage of manpower, and we had to deploy low-experience trainees to the emergency room; this hurt serving the patients because many of them weren't prepared to take care of critically ill patients. It happened several times that a patient was too ill, and they didn't realize how bad the condition was. They sent the patient for CT or ultrasound, where the patient fainted, was arrested, or whatever, and these were very problematic for us (M6).”

### 3.3. Threats to the Health of Medical Teams

The participants in this study believed that their job in the COVID-19 emergency department was a health threat to themselves and even their families, and problems would arise for them. Threats to the health of medical teams included two subcategories: anxiety and worry, and fear of contracting COVID-19. An example of class development process is presented in Table 2.

#### 3.3.1. Anxiety and Worry

Most of the participants stated that the COVID-19 peak was associated with several concerns for them. On the one hand, they were worried about providing equipment and medicine for the patients, and on the other hand, they were worried about the worsening of the patients' conditions leading to death. They were also concerned that their great efforts might be in vain due to the lack of follow-up care by discharged patients or the lack of quarantine of the people close to the patients. Other factors causing concern and anxiety among the participants included anger over the noncompliance with protocols by the public, feelings of injustice and inconsistency in the provision of equipment for all hospitals, and the stress caused by the deaths of patients.

“It was really upsetting and stressful to go to the hospital. We went there, but our patients were in a condition for which we couldn't do anything (M3).”

#### 3.3.2. Being under Pressure

According to the participants, the fear of being carriers and the possibility of transmitting the disease to their family members, and the fear of contracting COVID-19 and the death of colleagues, put them under a lot of stress. Their fear may stem from unknowns about the coronavirus's future.

“I'm afraid to hug my child when I go home.”

“My wife and I haven't seen our 15-month-old baby for about 48 days (M5).”

### 3.4. Fluctuations of Extremity in the Face of COVID-19

The main class of extremity fluctuations in the face of COVID-19 included three subclasses: delay in understanding the disease, overestimation, and underestimation. Based on the participants' experiences, people's functions varied at different times during the crisis. At a certain point, the fear of an unknown disease led to a surge in people visiting medical centers with unrelated symptoms. However, during the peak of the disease, the fear of hospital contamination led patients to either refuse to visit medical centers, even though they had symptoms that were completely related, or to refer themselves to a stage where there was no viable treatment available. Table 2 provides an example of how this class formed and includes quotes from the participants.

#### 3.4.1. Delay in Understanding the Threat of the Disease

Most of the participants emphasized the delay in understanding the disease by the public. Understanding the severity of the danger that threatens the individual is the driving force behind action. The participants found out that the more people felt threatened, the more careful they were in following prevention protocols. Perceptions of the risk of developing COVID-19 were influenced by factors such as knowledge, personality traits, postillness experiences of acquaintances, and their beliefs about the disease. According to the participants, nonobservance of protocols by people and holding celebrations, funerals, and gatherings; not taking the disease seriously until the number of deaths increased; the cultural challenge of believing late; and not believing the risk of infection until family infection occurred indicated a delay in understanding the threat of the disease.

“Until the end of the second wave of COVID-19, people still didn't believe it, and this caused the whole family to refer to the hospital after the infection (M3).”

“Patients with a positive PCR resist entering the gray area and say, Don't take us with COVID-19 patients (M8).”

#### 3.4.2. Overestimating the Risk of the Disease

According to the participants, the large number of visits with minimal symptoms at the onset of COVID-19, and the referrals of asymptomatic people or those with very few symptoms due to their irrational fear or obsession, indicated overestimating the risk of the disease.

“I had a lot of patients who came without even one COVID-19 sign (M1).”

“When they're told that they have COVID-19, they run away from the hospital and refuse to be admitted. In fact, they believe that we'll kill patients in the hospital. Our main problem is their escape and fear (M6).”

#### 3.4.3. Underestimating the Risk of the Disease

The participants stated that resistance to hospitalization in COVID-19 wards despite the worsening of the disease and the lack of referral of positive COVID patients with underlying diseases indicated an underestimation of the disease. The participants also said that patients with non-COVID diseases also faced the challenge of delaying primary treatment in these conditions because a long time had to be spent rejecting the diagnosis of COVID-19 by performing a test or CT scan.

“I myself encountered many of them who didn't bring their patients to the hospital. I mean, they tried as much as possible to treat the patients at home, and, well, many of them had risk factors and weren't treated properly. So, they referred to the hospital when the patient was critically ill (M2).”

### 3.5. Policymaking at the National Level

The last major class in this research was policymaking at the national level, with four subclasses including public awareness, intersectoral cooperation, planning and organizing human resources and equipment, and efficient management.

#### 3.5.1. Culture-Building with Appropriate Information Provision

According to the participants' experiences, culture-building through the use of an appropriate media environment was a factor that played a facilitating role in the process of controlling COVID-19. Timely provision of information, accurate statistics, mental health skills training, prevention and quarantine techniques, and stress management training will lessen the disruption of the COVID-19 management process. Thus, avoiding exaggeration in media reports would contribute to timely referrals of patients.

“The propaganda had caused a fear among people, and they referred to the hospitals unnecessarily (M)”.

#### 3.5.2. Intersectoral Cooperation

The participants had experienced that comprehensive cooperation and consensus of all government institutions were necessary for encouraging people to follow health protocols and organizing the people's jihadi services to provide required medical items.

“Solidarity, consensus, and proper management of resources, as well as making right decisions at the macro level of the Ministry of Health, etc., can minimize the challenges (M9).”

#### 3.5.3. Planning and Organizing Human Resources and Equipment

As the participants stated, establishing 24 hour and well-equipped field hospitals for COVID-19 patients, investigating errors in diagnosis and treatment, planning to provide needed medications, planning to follow up the patients during recovery, planning to quarantine carriers, planning to provide psychological support for the staff, planning to provide material and spiritual support for medical teams, and having a program to support the recruitment of manpower were all effective in controlling COVID-19.

“In general, budget, equipment, and manpower are the three important factors that can be managed and organized at the national level (M9)”.

#### 3.5.4. Efficient Management

Based on the participants' experiences, proper management (budget, equipment, and manpower), managers with medical knowledge, managers with relevant work experience, and supportive managers had an effective role in the process of controlling COVID-19. According to the data, fair management of supplying medicine and equipment, financing the treatment of COVID-19 patients, and using an appropriate incentive system for the medical team were other features of efficient management during the COVID-19 crisis.

“Skilled managers should manage the crisis. They should have work experience as well as scientific and work-related information. But sometimes we see, for example, a lawyer with no knowledge of sanitary ware and consumables become the head of the hospital; well, we won't expect the right things or successful results to be achieved in that hospital because the party that deals with the doctors doesn't know anything, doesn't have medical knowledge, and doesn't know about the consumables. When he's informed of a crisis, he doesn't understand at all! It's not up to his literacy at all (M10).”

## 4. Discussion

Health systems across developed and developing countries are under great pressure to limit the spread of coronavirus, and most of this responsibility is imposed on front-line health workers (medical emergencies) [10]. This article aimed to highlight some serious challenges that frontline health workers are currently facing and offer specific recommendations for reducing the burden imposed on them in order to ensure that the services provided are fast, well-equipped, and efficient. Analyzing the data from the experiences of the emergency medicine specialists participating in this study led to the extraction of four themes, including threats to the health of medical teams, structural environmental factors, fluctuations of people's extremities in the face of COVID-19, and finally, policymaking at the national level.

Structural environmental factors had two subclasses: equipment and human resources, and environmental factors. As in our research, other studies experienced a shortage of human resources and equipment [11]. Practical aspects of the job that should be considered for front-line health workers included appropriate personal protective equipment (PPE), goggles, and shorter working hours, all of which provided greater support and protection for nurses [12]. Alizadeh et al. proposed that the economic problems caused by the sanctions could be among the challenges considered by health workers that could be effective in the shortage and high cost of medicine [13]. In a study in India, 57% of the participants reported inappropriate ventilation, and adequate hand washing services were not available to patients in 12 locations. The lack of N95 masks, surgical masks, and gowns was also reported [14]. The lack of equipment not only for the staff but also for the patients was considered a problem, and the study by Phua et al. addressed the lack of hospital beds, ICU beds, and ventilators as well [15].

The shortage of human resources was another challenge caused by environmental factors in the present study. In the beginning of the pandemic, a considerable portion of expert health care providers refused to cover the COVID-19 wards, and this might have led to lower treatment quality [16]. Previous studies also confirmed that a lack of staff training on PPE and using and maintaining ventilator systems was a key issue in the management of epidemic diseases [17]. Increased workload was one of the reported complaints of the health care workers in this study, as was another one [18]. According to the results of a study, nurses who had a lot of workloads faced challenges in adapting to a new work environment, mastering equipment, and working with a new medical team [19, 20].

The threat to the health of medical teams was a theme, including concern about infection. One of its conceptual codes was concern about being a carrier and transmitting the disease to family members, which is consistent with the results of the study by Huang et al. [21]. Another challenge was related to the infection and death of colleagues, which is in line with the study by Sherin [22]. According to a study, nurses who used PPE for a long time had longer shifts and less sleep and avoided eating, drinking, and even using the toilets [23].

A study found that health workers experienced significant levels of anxiety, depression, and insomnia during the COVID-19 epidemic [24]. Similarly, concerns such as the stress of patient death, medical team's fatigue, feeling angry about people not following the protocols, fear of food contamination, stress of receiving protective equipment on time, feeling of hopelessness and despair with the death of patients, feeling upset about the deterioration of patients, an unworried follow-up system to track discharged patients, and worries about the nonquarantine of the people close to the patients and the futility of the efforts were common complaints in our study. Several studies have identified the social stigma of medical staff as a potential source of transmission [25]. In addition, as Sheng et al. stated, there was inequality in the workload and remuneration of the nurses and other medical staff [19].

The present research also showed nurses' dissatisfaction with their employment status, dissatisfaction with insufficient income and salaries, frustration due to a lack of medicine and equipment, feelings of injustice in providing equipment to hospitals, and a shortage of human resources and hospitalization space. Tallman et al. stated that unfair distribution reduced job satisfaction and willingness to work hard and commit to the organization [26]. Furthermore, high workloads caused nurses to suffer from physical and mental burnout, which was partly associated with their job satisfaction. This is in line with the present study [27].

In our study, fluctuation of people's extremities in the face of COVID-19 was a theme that referred to numerous problems: anxiety, being under pressure, overestimation or even underestimation of the risk, and delay in understanding the threat of the disease by the people. In another study, nonobservance of health protocols and recommendations by the people and their negligence caused health workers to worry about the further spread of the epidemic [13]. This section also raised concerns about the lack of segregation and quarantine following positive test results, which led to the spread of the disease within the community [28].

For better management of the COVID-19 epidemic, the four subgroups mentioned in the interviews were as follows: public awareness with appropriate information provision (accurate media atmosphere, accurate and timely information provision, public education—increasing people's knowledge about COVID-19, training on professional characteristics of medical teams, providing accurate statistics, training to deal with stress, avoiding exaggeration and overestimation of the disease, mental health skills training, proper prevention and quarantine training); intersectoral cooperation (comprehensive cooperation and consensus of all government agencies in addressing the challenges posed by the disease, cooperation of all government agencies in encouraging compliance with health protocols, coordination in addressing people's living conditions, coordination and synergy in society and all government agencies, organizing jihadi services to provide required health items); planning and organizing human resources and equipment (establishing 24 hour well-equipped field hospitals for COVID-19 patients, investigating errors in diagnosis and treatment, providing needed drugs, planning follow-ups for the patients during recovery, planning quarantine for carriers, psychological support programs for the personnel, material and spiritual support programs for medical teams, and medical team employment support programs); and efficient management (proper management (budget, equipment, and manpower), hiring managers with medical knowledge, wealthy and skilled management, managers with relevant work experience, penalties for people who do not follow health protocols, fair management of supplying sufficient medicine and equipment, financing the treatment of COVID-19 patients, and an appropriate incentive system for medical teams).

One study mentioned the following suggestions for better management of the COVID-19 epidemic: prioritizing health workers for diagnostic, supportive, and therapeutic services; providing (PPE); isolating and quarantining suspects and patients; and training, informing, and observing the protocols [11]. Another study suggested the following to prevent dysfunction: assessment of ICU bed capacity; ability to increase ICU beds with alternative care sites such as postanesthesia care units; preparation for patient separation and arrangement; provision of public information on ways of transmission; and following health recommendations [29]. Sherin suggested launching an appropriate screening system in each health center for COVID-19 patients, increasing the number of trained staff to reduce excessive stress and burnout, and providing specialized training on intensive care and other related specialties for physicians, nurses, and paramedics [22].

## 5. Conclusion

This study demonstrated the importance of anticipating the most acute scenario in critical situations to prevent surprises and health threats to the medical team. We must provide and maintain diagnostic and therapeutic equipment, medications, and personal protective equipment in a timely manner to deal with biological crises. Predicted shortages of skilled manpower need to be considered, and their scientific and skill weaknesses should be addressed in order to maintain crisis preparedness. We can prevent the spread of the virus by adopting appropriate management policies, which include culture-building, information provision, cross-sectoral coordination, planning and organization of human resources and equipment, and efficient management.

One of the limitations of this study is that it is not possible to generalize its results. Furthermore, the challenges and solutions expressed in this study are based on interviews conducted during the crisis response phase. After the crisis and the reconstruction phase, participants may gain other new experiences. We suggest that future research should review the published experiences of countries similar to Iran that are affected by this epidemic, compile a rich collection of emergency medicine specialists' experiences, and present a model for crisis management in infectious diseases.

## Data Availability

The data used to support the findings of this study are available from the corresponding author upon request.

## Abbreviations

EMS: Emergency medical services
PPE: Personal protective equipment.

## Supplementary Materials

Supplementary Materials Table 1: main classes, subclasses, and basic conceptual codes extracted from the data. Table 2: an example of class development process.
