# Supplementary material for: Exploration of the Challenges of COVID-19 from the Perspective of Emergency Medicine Specialists
Source: Emerg Med Int. 2024 May 25;2024:5536103. doi: 10.1155/2024/5536103 (PMC11144065; doi:10.1155/2024/5536103)
Supplement: Supplementary Materials — Table 1: main classes, subclasses, and basic conceptual codes extracted from the data. Table 2: an example of class development process. [file 5536103.f1.docx]

Table 1: Main classes, subclasses, and basic conceptual codes extracted from the data

| **Theme** | **Subclasses** | **Basic Conceptual Codes** |
| --- | --- | --- |
| Structural Factors | Deficit of Medical Equipment | Lack of perfect 24-hour CT scan machine |
|  |  | Lack of prevention facilities (masks, protective gowns, disinfectants, etc.) |
|  |  | Insufficient equipment for patients (masks, ventilators, medicine, etc.) |
|  |  | Lack of medication or delay in drug supply at the peak of the disease |
|  |  | Old oxygen generators and lack of oxygen |
|  |  | Drug supply problems due to economic sanctions |
|  |  | Lack of equipment (disinfectants, masks, oxygen, monitoring beds) |
|  |  | Lack of ventilators |
|  |  | High cost of medicine |
|  | Shortage of Human forces | Lack of manpower |
|  |  | Employing novice staff |
|  |  | High workload of manpower |
|  | Insufficient hospitalization space | Lack of physical space commensurate with the number of patients |
|  |  | Lack of distance in the hospital due to insufficient space |
|  |  | Lack of unoccupied beds during the peak of the disease |
|  |  | Common toilets for Covid and non-Covid patients |
|  |  | Hospitalization of suspicious and positive cases in the same physical space |
| Threats to the health of medical teams | Anxiety and worry | Medical team’s Fatigue |
|  |  | Feeling angry about not following the protocols by people |
|  |  | Worried about contaminated food |
|  |  | Concerned about timely receipt of protective equipment |
|  |  | Concerned about lack of medicine and equipment |
|  |  | Feelings of injustice and inconsistency in the provision of equipment for all hospitals |
|  |  | Concerned about the deterioration of patients |
|  |  | Stress caused by patient |
|  |  | Feelings of hopelessness and despair with the death of patients |
|  |  | Worried about the lack of a follow up system for discharged patients at home |
|  |  | Concerned about the lack of quarantine of the patient’s companions and the futility of the efforts |
|  | Being under Pressure | Fear of infecting family members |
|  |  | Fear of being a carrier |
|  |  | Fear of contracting COVID-19 and death of the medical team |
| Fluctuations of extremity in the face of COVID-19 | Delay in understanding the threat of the disease | Non-observance of protocols by people (holding celebrations, funeral ceremonies, gatherings, etc.) |
|  |  | death Irrational fear at the onset of an epidemic |
|  |  | Not taking the disease seriously until the end of the second wave |
|  |  | Cultural challenge of believing late |
|  |  | Not believing in the risk of infection until family infection |
|  |  | Patient resistance to hospitalization in COVID-19 ward |
|  |  | Fear and escape from hospitalization (denial) following a positive COVID-19 test |
|  | Overestimating the risk of the disease | High referral pressure of patients with minor symptoms at the onset of COVID-19 |
|  |  | Excessive obsession and referral with very few symptoms |
|  |  | Asymptomatic referrals due to fear |
|  |  | Delay in the main treatment of patients with non-Covid19 diseases due to the need to reject the diagnosis of Covid19 (CT scan and Covid19 test) |
|  | Underestimating the risk of the disease | Delay in referral due to fear of going to medical centers |
|  |  | Fear of infection by going to medical centers |
|  |  | Non-referral of patients with risk factors |
| Policy-making at the National Level | Public awareness | Accurate media atmosphere |
|  |  | Accurate and timely information provision |
|  |  | Public Education - Increasing public awareness of corona care |
|  |  | Training on professional characters of the treatment team |
|  |  | Providing accurate statistics |
|  |  | Stress management training |
|  |  | Avoid exaggeration and stigma (so that patients refer in time) |
|  |  | Mental health skills training |
|  |  | Proper training on prevention and quarantine methods |
|  | Inter-sectoral Cooperation | Comprehensive cooperation and consensus of all government institutions in addressing the challenges posed by the disease |
|  |  | Cooperation of all government agencies in encouraging compliance with health protocols |
|  |  | Coordination in addressing the people’s living conditions |
|  |  | Coordination and synergy in the community and all government agencies (in encouraging people to follow protocols until the provision of the equipment) |
|  |  | Organizing jihadi services to provide required health items |
|  | Planning and Organizing Human Resources and Equipment | Establishment of 24-hour well-equipped field hospitals for COVID-19 patients (lack of isolation rooms in public and private hospitals) |
|  |  | Investigation of errors in diagnosis and treatment (pitfall) |
|  |  | Supplying required medicines |
|  |  | Planning follow-up of patients during recovery |
|  |  | planning the quarantine of carriers |
|  |  | Planning Personnel Psychological Support |
|  |  | Material and spiritual support program for the treatment team |
|  |  | Medical team employment support program |
|  | Efficient Management | Proper management (budget, equipment, manpower) |
|  |  | Employing managers with medical knowledge |
|  |  | Rich and skilled management |
|  |  | Managers with relevant work experience |
|  |  | Penalties for people who do not follow health protocols |
|  |  | Fair management of supplying adequate medicine and equipment |
|  |  | Funding for the treatment of COVID-19 patients |
|  |  | Appropriate incentive system for medical team |

Table 2: An example of class development process

| A sample of participants' statements | Basic code | Subclass | Main class |
| --- | --- | --- | --- |
| It’s one year we’ve been involved, but they don’t comply. We ask them: did you travel? Yes; did you have a gathering? Yes; did you have a funeral? Yeah! Well, the result is getting COVID-19. It makes me feel angrier; (Participant number …)  We’re constantly worried about returning home or not? Should we quarantine ourselves or not? After the shift, should we return to our children and family or quarantine ourselves? (Participant number ...) | Feeling angry that people were not following health protocols  Worried about being a carrier | Anxiety and worry  Risk of infection | Threat to the health of medical team |
| They got afraid and referred unnecessarily.    I had a lot of patients who came without even one COVID-19 sign.  There were many patients with underlying diseases, but unfortunately, due to their fear and anxiety and the delay in referring to the hospital, they suffered irreparable complications and even death. | Irrational fear at the onset of the epidemic  Excessive obsession and referral with very few symptoms  Delay in referral due to fear of going to medical centers | Delay in understanding the threat of disease    Underestimating the risk of infection  Overestimating the risk of infection | fluctuations of people’s extremity in the face of COVID-19 |
